# Non-invasive online wavelength measurements at FLASH2 and present benchmark

**DOI:** 10.1107/S1600577517013893

**Published:** 2018-01-01

**Authors:** Markus Braune, Jens Buck, Marion Kuhlmann, Sören Grunewald, Stefan Düsterer, Jens Viefhaus, Kai Tiedtke

**Affiliations:** a Deutsches Elektronen-Synchrotron – DESY, Notkestrasse 85, 22607 Hamburg, Germany

**Keywords:** free-electron laser, soft X-ray, wavelength, photoionization, rare gases

## Abstract

At FLASH2, the free-electron laser radiation wavelength is routinely measured by an online spectrometer based on photoionization of gas targets. Photoelectrons are detected with time-of-flight spectrometers and the wavelength is determined by means of well known binding energies of the target species. The wavelength measurement is non-invasive and transparent with respect to running user experiments due to the low gas pressure applied. Sophisticated controls for setting the OPIS operation parameters have been created and integrated into the distributed object-oriented control system at FLASH2. Raw and processed data can be stored on request in the FLASH data acquisition system for later correlation with data from user experiments or re-analysis. In this paper, the commissioning of the instrument at FLASH2 and the challenges of space charge effects on wavelength determination are reported. Furthermore, strategies for fast data reduction and online data processing are presented.

## Abstract 2

The commissioning and the first year of operation of the online photoionization spectrometer OPIS at FLASH2 is reported.

## 1. Introduction   

For wavelength measurements in the vacuum-ultraviolet (VUV) and extreme-ultraviolet (XUV) range, optical diffraction gratings of various types are commonly used to disperse the radiation. In monochromator beamlines of accelerator-based photon sources, a spatial confinement by means of slits filters out fractions of the spectral distribution for applications which require narrow spectral bandwidths. With sophisticated designs, high resolving powers up to several 10000 can be achieved. By imaging the full dispersed beam using suitable screens such as Ce:YAG crystals and CCD cameras, these instruments can be used to observe the spectral distribution. In the classical layout, the detector unit is placed in the transmission direction, hence spectral measurements are invasive, *i.e.* the beam is blocked and not available for user experiments. This does not impose major inconveniences at synchrotron facilities, since the radiation properties are commonly assumed to be stable.

At free-electron laser (FEL) sources based on self-amplified spontaneous emission (SASE) such as FLASH (Ackermann *et al.*, 2007 ▸; Faatz *et al.*, 2016 ▸), however, the radiation properties fluctuate due to the stochastic nature of the SASE process. Hence, it is desirable to characterize these properties continuously in parallel with beam delivery to a running user experiment.

With respect to non-invasive wavelength measurements, it is favorable to use a grating spectrometer design in which the grating acts as an integrated optical element of the beam transport in the beamline, optimized to transmit most of the photon intensity in zeroth order, while the dispersed light of the first order is used for wavelength monitoring. Such a setup in combination with new-generation ICCD cameras with high frame rates or fast line detectors, such as the GOTTHARD detector (Mozzanica *et al.*, 2012 ▸), poses a promising concept for a grating-based online spectrometer capable of single-shot resolved measurements with up to MHz repetition rate (Palutke *et al.*, 2015 ▸).

A corresponding solution has been established at FLASH by supplying the existing variable-line-spacing grating spectrometer (Brenner *et al.*, 2011 ▸) with a GOTTHARD detector, enabling wavelength monitoring for the three beamlines of the FLASH1 BL-branch which is not equipped with a monochromator. Furthermore, a mobile grating spectrometer with the same type of detector is available for diagnostic purposes at the experimental stations.

At FLASH, several methods developed to measure radiation properties are based on atomic photoionization of gas targets. The gas monitor detector (GMD) (Richter *et al.*, 2003 ▸; Tiedtke *et al.*, 2008 ▸), for example, for measurements of the absolute pulse energy, is used at FLASH as well as LCLS, SwissFEL and European XFEL (Tiedtke *et al.*, 2014 ▸; Moeller *et al.*, 2015 ▸). In this context, the idea of using photoionization spectrometry of gas targets consequently came up as an alternative approach to online wavelength measurement (Wellhöfer *et al.*, 2008 ▸). A corresponding prototype device with electron and ion time-of-flight spectrometers has been developed and its applicability for wavelength measurements has been proven in extensive tests (Braune *et al.*, 2016 ▸). Based on these studies, an improved version has been incorporated in the photon diagnostics section of the FLASH2 beamline as a standard tool for routine wavelength measurements. Photoelectron spectroscopy with time-of-flight (TOF) analyzers is also applied as a diagnostic tool at the SASE3 undulator branch at the European XFEL implementing an enhanced polarimeter design (Buck, 2012 ▸).

In the following, a report on the commissioning as well as recent developments will be given. We will discuss ways to overcome the current performance limitations. Special emphasis is put on space charge effects, which turned out to be a problematic issue for wavelength monitoring in multi-bunch mode with high repetition rates and at high photon flux. Furthermore, first results from the principal component analysis of recorded photoelectron spectra will be discussed with respect to the foreseen applications in data reduction and towards real-time wavelength analysis.

## 2. The OPIS instrument   

The OPIS instrument consists of four electron time-of-flight (eTOF) spectrometers with z-stack microchannel plate (MCP) detectors and one ion TOF with a multiplier detector in a single vacuum chamber. Gas-phase targets such as noble gases and nitrogen can be introduced in a controlled way by means of a needle valve regulation system. The target is photoionized by the FLASH radiation and the wavelength can be determined by measuring the kinematic properties of the generated photoions and photoelectrons. In the ion TOF spectrum different charge states 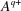 of the target gas show up at specific constant arrival times. The wavelength can be derived from intensity ratios of differently charged photoions.

In this paper we will concentrate on wavelength determination using the eTOF spectrometers. Here, the arrival time 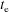 of the photoelectrons is measured with respect to the time 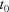 of arrival of the photon pulse. 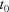 can be observed directly in the TOF spectra by means of a ‘prompt’ signal from Rayleigh scattering as well as fluorescence in some cases. The flight time of an electron, 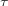 = 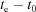, to travel from the interaction region to the detector reflects its kinetic energy 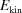. With the well known binding energies 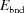 of the observed electronic orbitals of the target gas, the photon energy 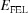 = 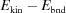 and consequently the wavelength 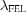 = 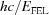 can be derived directly. Whereas the centroid of the photoelectron signals corresponds to the central wavelength of the FEL radiation, the shape of the photoemission line can be seen as a convolution of its natural line shape and the photon spectral distribution.

The four eTOFs are mounted in two pairs of opposing spectrometers perpendicular to the FEL beam. Changes in the photoelectron arrival time which are solely caused by beam position variations will have opposite sign in opposite spectrometers and hereby can be averaged out to avoid spurious interpretation as wavelength changes. All spectrometers are arranged in the ‘magic angle’ configuration, *i.e.* they are aligned at an angle of 54.7° with respect to the horizontal polarization axis of the FLASH radiation, so the measurement is independent of the angular dependence of the partial photoionization cross section (Hemmers & Lindle, 2001 ▸; Becker & Shirley, 1996 ▸). The chamber is made from μ-metal to shield the eTOFs from external magnetic fields which could distort the electron trajectories, especially for low kinetic energies, resulting in a reduction of electron spectrometer transmission and in errors of wavelength determination.

At a typical operating gas pressure of the order of 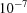
 mbar, the FEL photon transmission of the OPIS device is practically 100%. Within the photon energy range of FLASH2, the photoemission from the valence and first inner-shell electronic orbitals of the noble gases can be observed. Due to the different binding energies the resulting electron kinetic energy is mostly below 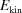 = 100 eV. 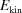 can be further reduced by applying a retardation voltage to the TOF analyzer to enhance the energy resolution.

The detector signals are recorded with fast analog-to-digital converters (ADCs) enabling single-pulse-resolved measurements in the FLASH2 burst-mode operation at bunch repetition rates up to 1 MHz. A more detailed description of the instrument, the operation principle and a report of the first experimental studies can be found in an earlier publication (Braune *et al.*, 2016 ▸).

### 2.1. Commissioning at FLASH2   

**Figure 1:**
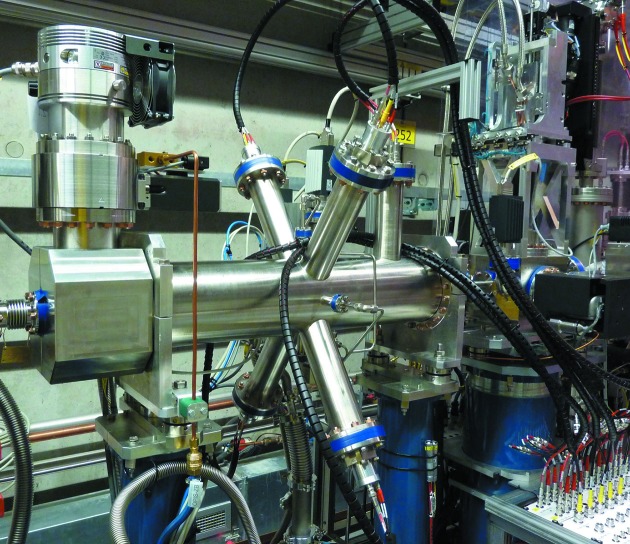
The OPIS instrument in the photon diagnostics section in the FLASH2 undulator tunnel. Four eTOF spectrometers are mounted in CF63 ports in the center of the basic CF160 tube of length 0.7 m. The spectrometers are aligned to the dipole plane perpendicular to the FLASH2 beam direction at an angle of 54.7° with respect to the horizontal radiation polarization vector.

The OPIS instrument was mounted in the diagnostics beamline in the FLASH2 tunnel next to the GMD intensity monitor of this section (Fig. 1 ▸). With four differential pumping stages which can bridge pressure differences of six orders of magnitude in total, a simultaneous operation of the two gas-based instruments using different target species can be ensured without gases mixing in either instrument.

In the commissioning phase the control devices of the operation components like target gas pressure regulation as well as MCP detector and retardation voltage supplies have been integrated into the distributed object-oriented control system (DOOCS) of FLASH (Ko & Chou, 2008 ▸). Data acquisition and transfer hardware have been tested and the analysis software of the prototype studies has been enhanced and connected to the DOOCS system as well. Most importantly, however, was the calibration of the OPIS device for different retardation voltages. For this, no external reference such as, for example, a grating spectrometer has been used, but the OPIS could be calibrated by means of Auger processes. Auger electrons possess constant kinetic energy arising from an internal electron rearrangement in the target gas atom after emission of an inner-shell electron in the ionization process. Therefore, Auger electrons can serve as fixed kinetic energy markers in the photoelectron spectrum. The well known Auger energies of the prominent xenon 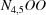 and krypton 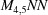 transitions (Werme *et al.*, 1972 ▸; Carroll *et al.*, 2002 ▸) in the range from 8.3 eV to 36.4 eV and 24.0 eV to 56.5 eV, respectively, could be attributed directly to the TOF values of the respective Auger lines using arbitrary FEL photon energies above the Xe 4*d* and Kr 3*d* thresholds. In additional measurements, the FLASH2 undulator gap was tuned precisely to photon energies at which the Xe 4*d* and Kr 3*d* photoelectron lines, respectively, exactly matched certain Auger line TOF positions. In this case, the photoelectron kinetic energy equals the corresponding Auger energy and hence the photon energy is determined. Consequently, the kinetic energies of the faster outer-valence electrons Xe 5*p*,5*s* or Kr 4*p*,4*s* are known as well, and the values together with the corresponding flight times can be added to the calibration data set. This self-calibration capability is a valuable property of the OPIS spectrometer. For operation at photon energies above the Auger threshold, the accuracy of the spectrometer’s energy scale can be confirmed at any time during the actual wavelength measurement by evaluating Auger line positions.

**Figure 2:**
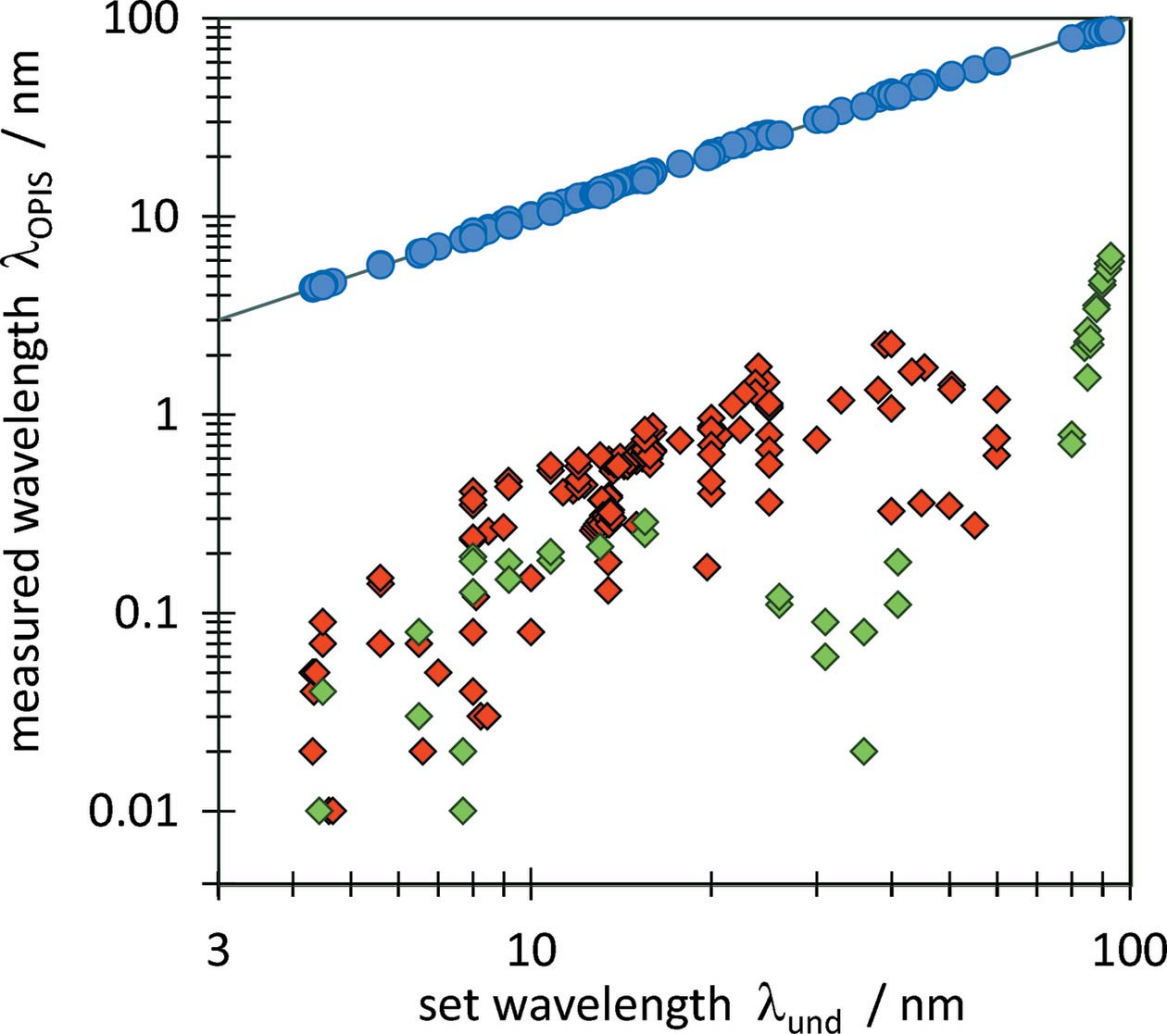
Compilation of 162 OPIS results 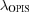 (blue circles) of wavelength measurements for different nominal FLASH2 wavelengths 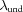. The deviation 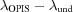 is plotted as absolute values (diamonds) in red and green colors for negative and positive sign, respectively.

After collecting a sufficiently large calibration data set, the operation phase was started for regular internal FLASH2 machine studies and first runs of user experiments. Fig. 2 ▸ shows a selection of OPIS results for various nominal wavelength values 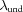 set with the undulator parameters, covering the complete FLASH2 range from 4.2 nm to 90 nm. The deviation 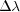 = 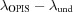 between measured and nominal wavelength shows different values for different measurements at the same or similar wavelength. 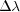 increases from 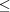
0.1 nm at the shortest wavelengths to several nanometers at the upper end of the FLASH2 wavelength range. The undulator gap setting for a certain nominal wavelength depends on the energy of the electrons in the FLASH accelerator which can be derived either from the radiofrequency of the superconducting accelerator cavities or the electron trajectories in a dipole magnet section. Recent studies revealed predominantly results with 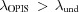 for the dipole-based and 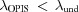 with better agreement for the RF energy-based gap settings. However, in both cases the deviation is considerably large and the results shown in Fig. 2 ▸ indicate a strong influence of various machine parameters which are set during or result from the SASE setup procedure. This demonstrates the need for wavelength measurement for setting up the machine to the desired wavelength for user experiments.

### 2.2. Energy resolution and wavelength determination accuracy   

TOF spectrometers of different types have been used for decades in photoelectron spectroscopy at synchrotron light sources. Various designs of the conventional linear spectrometer with electronic lens assemblies for photoelectron deceleration can be found in the literature, including demonstrations of the instrument performance in terms of the energy resolution; see, for example, Hemmers *et al.* (1998 ▸).

From a conceptual point of view, a basic guideline figure for the resolution of an eTOF spectrometer is determined by its length *l* and the opening angle α defined by the size of the electron detector area. Assuming a theoretical point source of the photoelectrons, the flight-path length of an electron reaching the detector can have values between the shortest distance *l* to the detector center and the longest distance 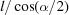 to the outer detector surface area. The corresponding TOF uncertainty 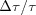 = 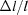 = 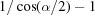 gives rise to a fundamental energy resolution 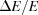 which is constant over the kinetic energy. With our type of spectrometer (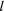 = 309 mm, 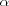 = 5°, 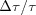 = 0.1%), this basic resolution is around 0.2% corresponding to a resolving power of 500. By applying retardation potentials, the resolution can be considerably increased. The resolution then depends on the kinetic energy, *i.e.* it is higher for low kinetic energies. With retardation, theoretical resolving powers can then be as high as a few 10000.

However, in an experiment the resolution is limited by a number of factors, such as the finite size of the interaction region (for example, defined by the spot size of a focused beam at an experimental station), broadening effects due to the electronic lens potentials and jitters of the light pulse arrival time and the trigger signal, but also the method of signal detection.

In experiments at synchrotron storage-ring photon sources, common single-event-counting techniques are used such as time-to-digital converter-based data acquisition systems employing constant fraction discrimination. With this method, the influence of the detector response function on the energy resolution is reduced to the steepness of the raising edge (typically a few hundred picoseconds for MCP detectors) and the temporal shape of this function is basically irrelevant. In such experiments an energy resolution of 1000 could be achieved with the type of eTOFs used in OPIS, at an experimental station with a focal spot size of 100 µm and using an eTOF retardation voltage of 25 V.

At FLASH2, the conditions for the wavelength measurements differ from the typical conditions at storage rings in two important aspects:

(i) The OPIS instrument is located in the photon transport beamline about 16.8 m behind the end of the undulator section. At this position the FEL beam diameter (FWHM) is ∼1 mm for shortest and ∼5 mm for longest wavelengths. Hence, photoelectrons from different starting points within a large acceptance volume can reach the detector and consequently the spread of possible flight paths and flight times is larger compared with the case of an interaction region defined by a focused beam.

(ii) The ADC traces of the analog MCP signals contain the temporal width of the detector response function which is defined by detector properties, such as the applied voltage and the aspect ratio l/*d* of the length *l* and the diameter *d* of a single channel of the MCP (Wiza, 1979 ▸). As a result the electron line profiles in the spectrum are convolutions with contributions from distributions of the electron kinetic energy and the trajectory length, as well as the detector response function. Depending on the FEL pulse energy and the wavelength, typically some 10 to 100 impacting electrons per shot build up a photoelectron or Auger electron line feature in the spectrum.

Both aspects give rise to considerable line broadening and reduction of resolution which primarily affects the determination of the spectral distribution from the photoelectron line shape. With respect to monitoring of the central wavelength, however, this broadening will not significantly reduce the eTOF performance, since for the central wavelength only the mean arrival time, *i.e.* the peak center position, of the photoemission line is relevant.

**Figure 3:**
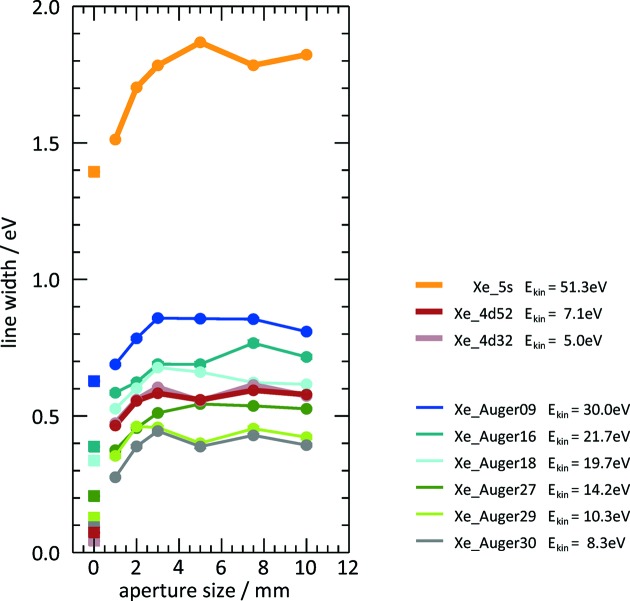
Plots of photoelectron and Auger line widths derived from an energy-converted xenon spectrum recorded at 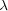 = 16.6 nm and 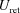 = 0 V, averaged from 600 FEL shots, as a function of the size of the aperture in front of the OPIS instrument. Values are absolute experimental line widths (circles) and calculated line width corresponding to the temporal width 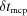 of the MCP detector (squares). All data points are mean values of the widths from the four eTOF spectra, weighted with the width error. The kinetic energies of the photo- and Auger electrons are given in the legend. Numbers in the Auger line designation correspond to the notation of Werme *et al.* (1972 ▸). From the Xe 4*d* signals an upper limit for the FLASH2 spectral width 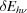 = 0.5 eV can be derived.

The influence of the two mentioned broadening effects has been exemplary illustrated in a study with xenon at a wavelength of 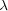 = 16.6 nm, in which the beam size was varied by means of the size of a beamline aperture in front of the OPIS instrument. Values of the width of photoelectron and Auger lines have been derived from spectra which have been averaged over 600 FEL shots and converted from TOF to kinetic energy scale. Fig. 3 ▸ shows the results for various single-component features which appear in the spectrum at different kinetic energies given in the legend.

The dependence on aperture size can be seen in the increase of the line widths for aperture sizes 1, 2 and 3 mm. A further opening of the aperture does not significantly change the width for all observed features. This indicates that the photon beam diameter in this measurement was about 3 mm for 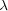 = 16.6 nm, which is a typical value. In relative terms the effect is quite similar for all photoemission lines.

The natural width of the Auger lines is of the order of some 100 meV, whereas the measured values are clearly larger and increase with the Auger kinetic energy. This is mainly due to the fact that the width of any Auger or photoelectron line 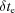 in the TOF spectrum cannot be smaller than the temporal width 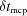 of the MCP detector signal. The relative proportion of 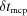 in the total width decreases with τ due to *t* ≃ 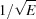 and dominates at very short flight times and high kinetic energies. From the prompt signal and fast photoelectron lines we could deduce 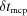 to be 1 ns. The values of the energy width 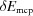 corresponding to solely 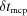 are given by the squares plotted at aperture size 0 mm, equivalent to hypothetical photoelectron or Auger electron lines without any energy width or geometrical and beam size broadening. These values agree fairly well with widths that result from propagating the measured curves to an aperture size of 0 mm.

The photoelectron lines Xe 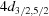 and Xe 5*s* contain the spectral width 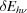 of the FEL radiation in addition. To determine the size 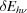 we consider the measurements with the smallest available aperture size of 1 mm to minimize the broadening effects. For Xe 5*s*, the total width may still be governed by 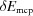 due to the high kinetic energy. However, for Xe 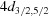 the width 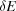 is considerably larger than the width of line ‘Auger30’ at a similar kinetic energy. This indicates that 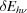 is the predominant contribution to the total width 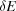. Hence, the measured value of approximately 0.5 eV can be regarded as the upper limit of 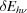 corresponding to 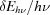 = 0.7%, which is a typical value for FLASH2 operation, as has been confirmed with the grating spectrometer at the FLASH2 beamline FL22 (Tanikawa *et al.*, 2016 ▸).

Xenon 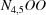 and krypton 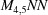 Auger spectra recorded for different operation conditions have been evaluated with respect to the ability of the eTOF to resolve neighboring Auger lines. We found that a resolving power for the electron kinetic energy 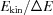 of 100 or better can be easily achieved for small beam sizes. However, the resolving power with respect to the photon energy 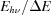 can be larger, especially at short wavelengths where one can take advantage of the lower electronic orbitals with large binding energies. For example, in a measurement at a photon energy 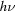 = 290 eV with argon the kinetic energy of the Ar 2*p* electrons is about 40 eV. With 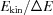 = 100, the absolute resolution 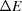 is 0.4 eV, which corresponds to 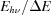 = 725 in this case.

**Figure 4:**
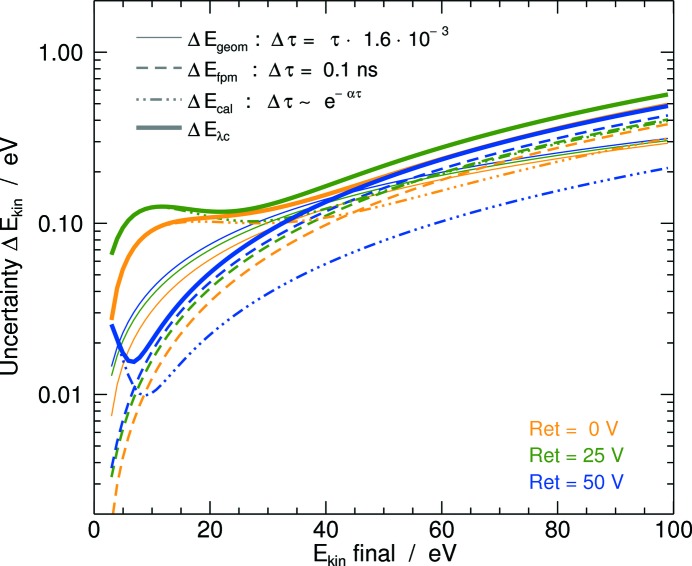
Uncertainty 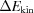 of the kinetic energy of detected photoelectrons arising from the uncertainty 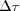 of the flight time, estimated by solely the acceptance angle of the spectrometer (thin solid lines), the error of the peak position parameter of the photoelectron line profile fitting procedure (dashed lines), and the residuals of the time-to-energy conversion calibration fit function with respect to the calibration input data (dash-dotted line), which have been adapted to an exponential function of the kinetic energy. The thick solid line represents the combined experimental uncertainty regarding the determination of the central FEL wavelength. The results are plotted in different colors for three retardation potentials 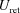 of 0 V, 25 V and 50 V *versus* the final kinetic energy 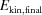 = 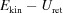.

The acceptance angle of the OPIS eTOFs is defined by the effective MCP detector area diameter of 28 mm and the entrance aperture of 3.2 mm at a distance of about 28 mm from the nominal beam center. Geometrically, this results in an acceptance diameter of about 6 mm at this distance and a TOF uncertainty of 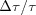 to 0.16%. The corresponding expected uncertainty 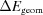 of the kinetic energy of the photoelectrons is plotted over the final kinetic energy 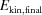 = 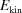 − 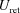 for different retardation voltages 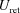 in Fig. 4 ▸. For a final kinetic energy range of 0 to 100 eV, 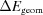 increases from about 0.01 to 0.2 eV.

To estimate the experimental uncertainty of a wavelength determination, we consider the error of the photoemission line peak position of the model function used for the spectrum line profile fit procedure. For this purpose, a data set of over 100 acquisitions for each of the four electron spectrometers has been evaluated containing over 1300 line fits of photoelectron and Auger signals spreading over a large TOF range. In the typical flight time interval of 90–180 ns, the mean value of the position fit-parameter error 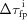 is about 0.04 ns with a standard deviation of 0.04 ns. We assess a constant error 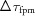 of 0.1 ns for which 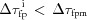 in 94% of the cases.

Furthermore, the residuals of the calibration function for the time-to-energy conversion, giving the deviation of this function from the calibration experimental input data, can be regarded as a measure of the absolute accuracy of the wavelength scale. The residuals show a decreasing trend along the TOF axis which can be approximated by an exponential function 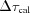 ≃ exp(−ατ). The corresponding uncertainties of the kinetic energy 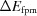 and 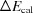 are plotted in Fig. 4 ▸ together with the expectation 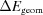. The results are consistent; however, the uncertainty of the calibration clearly dominates for low kinetic energies. We determine the combined experimental error for central wavelength determination, 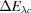, derived from 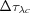 = 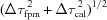.

For values 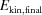 below 30 eV, the error 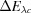 is smaller than 0.13 eV. Consequently, the relative uncertainty of the determined central wavelength 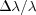 ranges from 9 × 10^−3^ at 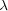 = 90 nm to 4 × 10^−4^ at 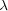 = 4.2 nm. Of course, from all the electronic orbitals available at a photon energy 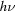 and with 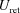, normally the photoelectrons with the lowest kinetic energy are chosen for wavelength determination. With the target gas species Ar, Kr and Xe and 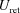 = 0, 25 and 50 V, indeed 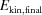 < 30 eV can be achieved over the complete FLASH2 wavelength range, except for the interval between 4.9 and 5.5 nm in which the uncertainty increases up to about 0.25 eV. Additional calibration data for 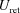 = 170 V, which is currently being established, will improve the accuracy for this wavelength range.

## 3. Wavelength monitoring operation   

**Figure 5:**
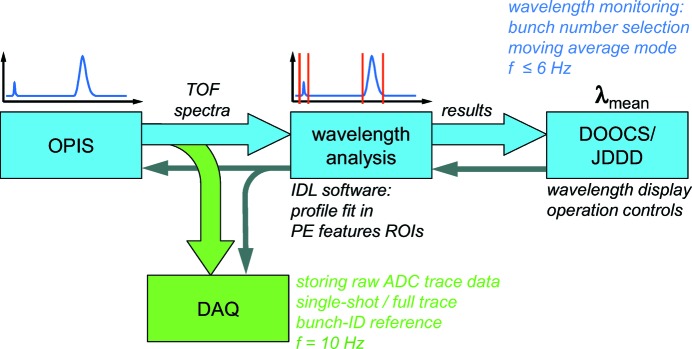
OPIS data acquisition scheme. Blue colors indicate the wavelength monitoring operation using moving average spectra of one arbitrarily selectable bunch of the FLASH2 pulse train. Data storing of single-shot resolved raw spectra (green) is available independently from monitoring operation. Panels implemented in the FLASH control system DOOCS are used to set the hardware parameters as well as to display the measurement and wavelength analysis results (gray). According values are continuously stored in the FLASH DAQ system.

A scheme of the current data acquisition and evaluation during OPIS operation is shown in Fig. 5 ▸. The wavelength analysis software has been implemented in the Interactive Data Language (IDL) programming environment and runs on a standard PC workstation. For online wavelength monitoring, only the spectrum of one bunch of the FLASH2 pulse train is recorded. The wavelength analysis is applied to a moving average of a certain number of latest single-shot spectra.

The analysis of the currently active average spectrum by means of a peak profile fit procedure is performed for one or more photoelectron features and for each of the four eTOF spectrometers. The corresponding individual results as well as the mean wavelength value are stored together with the actual OPIS operation parameter settings such as gas pressure, detector and retardation voltages in the FLASH data acquisition (DAQ) system. Due to the fit procedure, the analysis cannot keep up with the 10 Hz repetition rate of FLASH. Depending on the number of photoelectron features analyzed, sustained analysis rates of 1 to 6 Hz can be achieved. Nevertheless, this is completely sufficient as a feedback for wavelength tuning during FEL set-up and for quick wavelength adjustments or scanning schemes for user experiments.

If the user experiment requires single-shot resolved information, a corresponding mode can be activated in which, simultaneously to the monitoring operation, raw data traces of complete bunch trains are written directly into the DAQ system at the full speed of 10 Hz. These data can be processed in an offline analysis using the same software routines used for monitoring, customized or user procedures. The OPIS raw data traces are uniquely labeled with the serial FLASH pulse-ID number for correlation with other FLASH machine and photon diagnostics parameters or user data.

Currently, the MCP detector signals are recorded both with an oscilloscope (LeCroy WR625Zi, 10 GS s^−1^, 2.5 GHz, 8-bit) and MTCA ADC cards (SP Devices ADQ108, 7 GS s^−1^, 2 GHz, 8-bit). For wavelength monitoring either source can be used, but only MTCA ADC data are stored in the DAQ system if activated. Oscilloscope traces can be stored to the hard drive of the OPIS analysis computer in schemes of acquisition sessions, in which the set of *n* single-shot traces and the according average spectrum of the actual bunch position is saved together with parameters of the wavelength analysis procedure, such as setting of the regions of interest (ROIs) for prompt signal and photoelectron lines. This is mainly intended to be used for calibration confirmation, instrument studies and further tests.

### 3.1. Operation controls, panels and tools   

The target gas pressure level for operation is regulated by a motorized dosing valve (Pfeiffer EVR116) in combination with a control unit (Pfeiffer RVC300). Corresponding panels for remote actuation are integrated in the DOOCS system. The valve is mounted closest to the OPIS chamber between the gas inlet needle and a stainless steel tube switch board which forms the conjunction of supply conducts from a cabinet containing gas cylinders with the different target species. Gas changes and purging are performed in automated procedures by a combined programmable logic controller (PLC) for the gas inlet systems of the three gas-based diagnostic tools OPIS, GMD intensity monitor and attenuator. These procedures can be started and controlled by separate PLC panels which are not part of the DOOCS system.

For the high voltage supply for the MCP detectors and the retardation potentials for the different spectrometer flight-tube sections a modular multi-channel system (ISEG EHS8040 and EHSF005 modules) has been chosen. Voltage values as well as limits are set and read-back values are displayed in the DOOCS system using a CAN-bus interface.

The information displays and controls for OPIS operation are structured in DOOCS panels of different levels of detail. We will highlight the most important features in the following.

In the OPIS main panel the wavelength results for each eTOF and the mean wavelength value are permanently displayed and updated. Here, the number *n* of single-shot spectra to be averaged for analysis can be set at any time to arbitrary values including 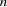 = 1 for single-shot analysis. Typically, *n* is of the order of 50. Furthermore, the number of the bunch to be analyzed can be chosen. A corresponding trigger delay is calculated and applied to the back-plane trigger signal of the MTCA crate by the timer card, which shifts the recording time window of the ADCs as well as the oscilloscope to the requested bunch position. Retardation voltages can be changed for all eTOF spectrometers equally or individually. In all cases the monitoring operation continues after refreshing the average spectrum buffer.

At start-up of the operation, default photoelectron features are used for wavelength determination, which is normally the electronic orbital of the actual target gas with the lowest kinetic energy available with the currently applied retardation voltage. However, different photoelectron lines can be chosen *via* a selection panel. The accurate positions of the ROIs containing the photoelectron lines have to be confirmed and adjusted if necessary in an interactive dialog before start of operation (*cf.* §2.1). During operation the ROI positions in the TOF spectrum are automatically adapted on wavelength changes by undulator movement or after switching to a different retardation voltage to improve accuracy.

For visual inspection, actual recorded spectra and anlysis fit curves can be viewed in the IDL environment. In addition, raw data traces of the MTCA ADCs are displayed in DOOCS diagram panels.

**Figure 6:**
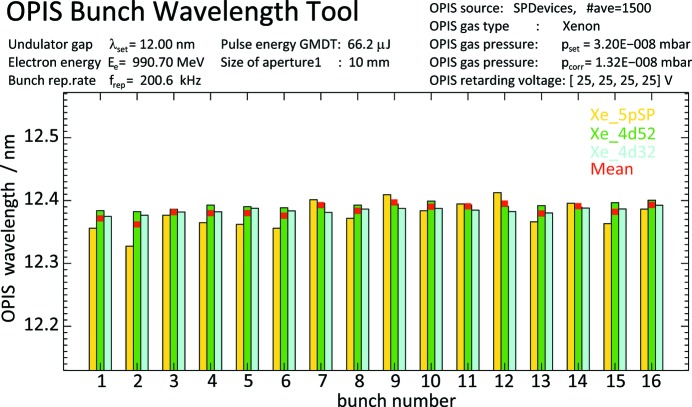
A special tool calculates and plots the wavelength results for each bunch in the FLASH2 pulse train in the form of a histogram for visualization of systematic wavelength changes along the bunch number. The example shows the result for operation at a set wavelength 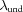 = 12.0 nm with trains of 16 bunches. The OPIS measurement was made with xenon using three photoelectron features Xe 5*p*, 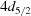 and 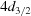 and a retardation voltage of 25 V. The individual as well as the mean wavelength values are plotted and show a flat distribution.

In monitoring mode the differences in the FLASH2 wavelength of different bunches in the pulse train can be revealed by switching the actual bunch number in the main control panel. For more efficient investigation of systematic changes of the wavelength along the pulse train, a special tool was developed which can be used independently of and in parallel with the running monitoring operation. It sums up a preset number of successive traces of complete bunch trains from the MTCA ADC system, dissects the average trace into spectra of individual bunch numbers and performs the wavelength analysis sequentially for each bunch. An output plot of this tool is shown in Fig. 6 ▸.

### 3.2. Space charge effects   

A major challenge for OPIS wavelength measurements can be the space charge accumulated in the interaction region in the process of photoionization. Due to the high intensity of the FEL radiation the effect on the photoelectrons can be substantial. The positive charge of the only slowly moving heavy ions decelerates the photoelectrons and hence falsely leads to longer wavelength results.

**Figure 7:**
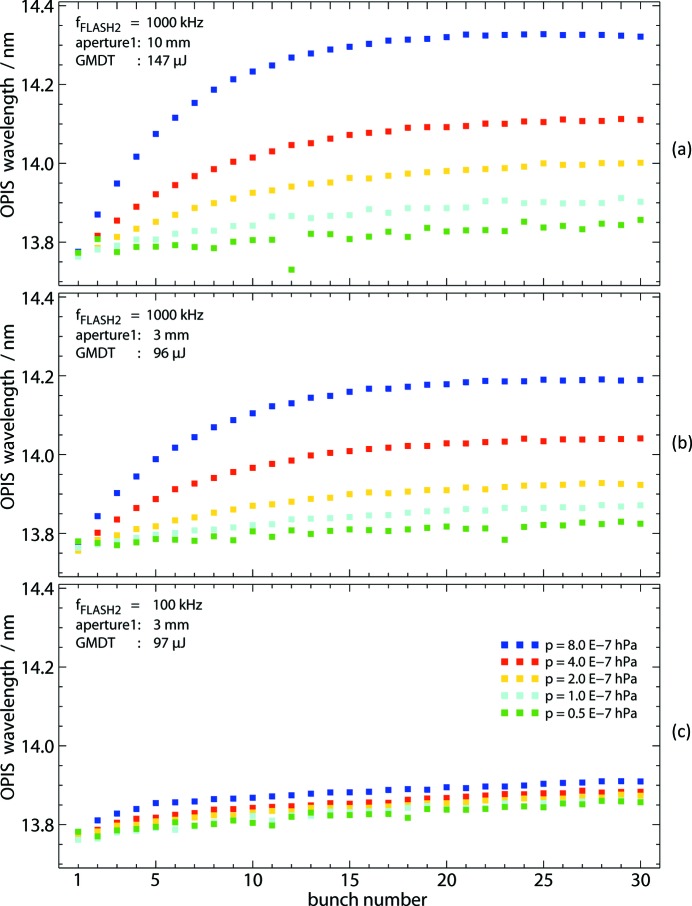
The effect of space charge on measured wavelength as a function of target gas pressure, FLASH2 repetition rate and beam size. The panels show wavelength results for the 30 bunches of the pulse train at 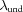 = 13.5 nm for different target gas pressures. (*a*) A considerable wavelength shift increasing over the pulse train is found at high repetition rate of 1 MHz and a large aperture (uncropped beam). (*b*) Reduced space charge effect when the beam size is reduced by means of a smaller aperture. (*c*) Almost no space charge effect if, in addition, the repetition rate is lowered to 100 kHz. The residual slope of λ along the bunch train in the curves for 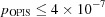 reflects a real trend in FEL wavelength. For all measurements the bunch wavelength tool described in §3.1 has been used.

In order to assess the magnitude of the effect and its dependence on the crucial parameters such as target gas pressure level and pulse repetition rate, space charge has been investigated in dedicated studies. As an example, some of the results are depicted in Fig. 7 ▸.

Fig. 7(*a*) ▸ shows OPIS results of measurements with argon at 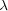 = 13.8 nm and a bunch repetition rate of 1 MHz. The aperture in front of the OPIS instrument was set to 10 mm, not clipping the beam with a diameter smaller than 5 mm. The wavelength values for 30 bunches of the pulse train are plotted for various pressure levels. There is an almost linear increase in the beginning of the pulse train, where the ions created in successive bunches apparently add up leading to a growing space charge. The wavelength shift reaches a saturation value after approximately ten bunches indicating an equilibrium state between the rates of ions escaping the interaction region due to Coulomb repulsion and newly generated ions.

The maximum wavelength shift in the saturation regime is 0.5 nm for the highest target gas pressure of 8 × 10^−7^ mbar in this example. As expected, the shift is proportionally smaller for lower pressures and the effect in general is weaker for smaller beam sizes [see panel Fig. 7(*b*) ▸], for which the photon pulse energy is lower as well as the extension of the positive ion charge distribution being smaller.

In the measurements with a repetition rate of 1 MHz the saturation is reached between bunch numbers 10 and 15. Apparently, the argon ions need some 10 µs to leave the interaction region. This is corroborated by the measurement with a repetition rate of 100 kHz, shown in Fig. 7(*c*) ▸, for which there is almost no space charge effect and the wavelength values for a pressure up to 4 × 10^−7^ mbar are basically identical. The slight monotonic increase of λ along the bunch train in these curves is the signature of a real FLASH2 wavelength trend present for the SASE parameter settings at that time.

Note that the wavelength results of the first bunch are identical within the unit’s measurement error in all cases and are hence not biased by space charge. At this point, any previously accumulated ion charge has dissipated during the time of 100 ms between the pulse trains, and the first photon bunch always interacts with a neutral target. We conclude that the individual ionization processes in the first bunch are independent in a sense that each of the relatively fast Ar 3*p* electrons is not affected by the charge created from other ionization events of the same bunch.

For lowest possible operation pressure close to the base pressure of about 3 × 10^−8^ mbar the space charge is negligible even at the maximum repetition rate of 1 MHz and with the largest aperture size in certain cases as shown in Fig. 7 ▸. In this example, for 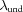 = 13.5 nm, the comparably small argon photoionization cross section of about 1 Mbarn is advantageous. Similar conditions can be established at different wavelengths choosing other target gas species. However, at long wavelength ≳45 nm the cross section for all noble gases is of the order of 10 Mbarn which means that space charge effects are difficult to avoid in this region. In addition, for photon energies above the Auger threshold, the mean charge per ionization event increases to values >2 which intensifies the space charge effect.

Even with a substantial space charge signature, relative changes of the wavelength and its trends can still be derived from the post-saturation region of the bunch train. Further systematic measurements could help to verify a computational model of the space charge effect which in turn would help to correct OPIS wavelength results measured under these conditions. Provided that the photon energy is above the Xe 4*d* or Kr 3*d* threshold, Auger lines still mark the absolute energy scale even in the presence of space charge, opening an opportunity for a measurement-based online correction.

However, there is also a number of technical upgrade options to eliminate space charge effects. One quickly implementable solution is to increase the pumping speed of the OPIS chamber with the goal of reducing the background pressure and hence allow for a lower target gas operation pressure. Secondly, electrodes for ion extraction by a pulsed electric field can be adopted with the existing apparatus and are deemed more effective. A further, more elaborate, approach being discussed in the community is the replacement of the effusive target gas beam by a supersonic jet assembly with which the ions would leave the interaction region after ionization just due to their high kinetic energy and aligned momentum.

## 4. Efficient DAQ storage of full bunch train information   

As mentioned in §3, the recorded ADC bunch traces of the eTOF detector signals for each FLASH2 pulse can be written to the FLASH DAQ system on request. Having data from four eTOF spectrometers sampled at a rate of 7 GS s^−1^ for a total duration of several hundred microseconds per pulse train and a pulse repetition rate of 10 Hz, saving complete traces would result in excessive data transfer rates of the order of some 100 MB s^−1^ and demand extensive amounts of storage space. Two methods concerning data handling are applied which are described in the following.

### 4.1. Grouping scheme   

**Figure 8:**
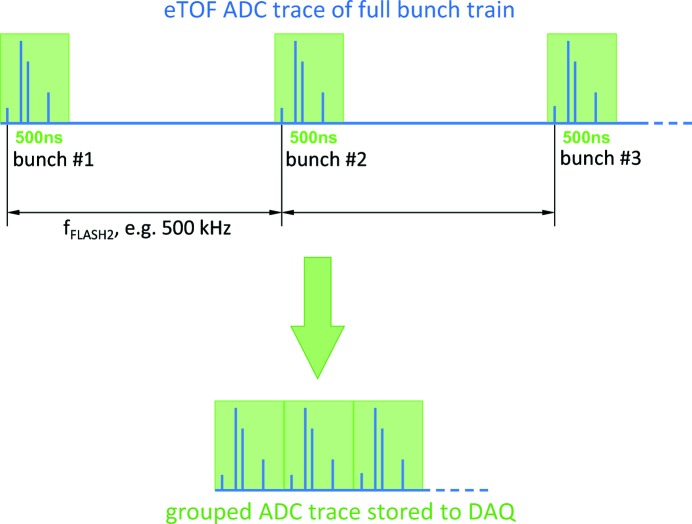
From the complete trace of the train, a grouping scheme cuts out intervals containing the eTOF spectra of all bunches. The condensed ADC trace containing only the relevant information is forwarded to the FLASH DAQ system.

Since the actual TOF spectra from the pulses span only a few hundred nanoseconds each, major parts of the ADC trace contain no information. Hence, a grouping scheme is used to select only the parts containing the TOF spectra and merge them to a condensed trace which is forwarded to the FLASH DAQ system (see Fig. 8 ▸).

The grouping parameters (number of blocks and spacing) can be entered *via* the ADC DOOCS panel. Obviously the number of blocks normally equals the number of bunches per train and the spacing between blocks is given by the FLASH bunch repetition frequency. The position of the individual blocks in units of samples is calculated automatically according to the sampling frequency and the FLASH bunch repetition rate. A spectrum block typically spans an interval of 3500 samples, corresponding to 500 ns. This value may be adapted to the TOF of the slowest electrons in the spectrum, providing a reduction of the raw data rate by a factor of ≥2 at 1 MHz or ≥20 at 100 kHz. The condensed ADC trace can be viewed in a plot panel for verification of the settings.

### 4.2. Fast data reduction for online analysis   

In contrast to traditional experiments in electron spectroscopy where the acquisition time is simply adjusted to the available count rate, the accumulation of data over an extended period of time is prohibitive when performing photon diagnostics with the aim of characterizing single pulses from a statistically fluctuating source such as a SASE FEL. From a data analysis perspective, one ends up here with large amounts of (single-shot) spectra with a rather low individual quality instead of a single spectrum with practically eliminated statistical uncertainty. In order to achieve a meaningful analysis in this scenario, we can exploit the fact that the parameters of interest (here, spectral center of mass and width) reveal only small fluctuations from pulse to pulse, so each spectrum can be analyzed in the context of all repetitions acquired. The scenario as outlined qualifies for the application of a principal component analysis (PCA) (Pearson, 1901 ▸; Jolliffe, 2002 ▸). In this section, we demonstrate how a PCA-based approach for photon diagnostics can lead to substantial gain in speed along with a dramatic reduction of the data volume handled.

PCA basically maps sets of correlated, multivariate data points (here, the eTOF spectra) into a lower-dimensional, orthogonal basis with uncorrelated components. We may interpret the basis of that space as a set of orthogonal functions (sampled at discrete points) used to expand the deviation of an individual spectrum from the average spectrum. As the basis is chosen to match the principal axes of the distribution of data points in descending order of variance along its direction, truncating the expansion after only a few terms effectively segregates features representative of the entire set of spectra from non-representative ones which have no correlation inside the statistical sample of all spectra taken, *e.g.* noise and counts not representing features of the ‘true’ spectrum.

From its representation 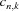 in the truncated basis 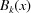 together with the mean spectrum 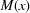, the approximation of a single-shot spectrum *n* in the original data space is given by a linear combination, which we refer to as reconstruction 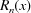,
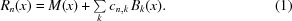
The potential of the PCA technique in this field was already demonstrated with measurements at the Variable Polarization Soft X-ray beamline P04 at PETRA III with an instrument akin to OPIS which also employs multi-channel eTOF spectroscopy. There, it serves as a general diagnostic tool, enabling the simultaneous observation of beam properties such as polarization and photon energy as well as beam position (Buck *et al.*, 2012 ▸). In analogy to the OPIS, studies using this apparatus at FEL sources have been reported recently (Allaria *et al.*, 2014 ▸; Lutman *et al.*, 2016 ▸).

**Figure 9:**
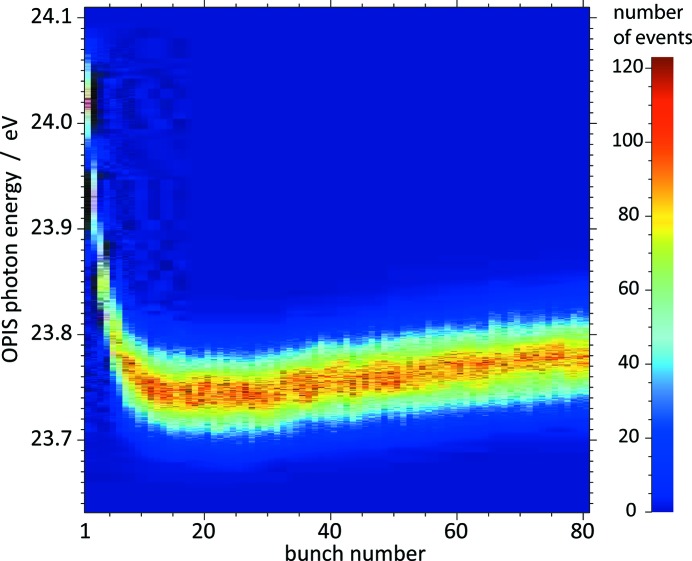
Two-dimensional histogram of the measured photon energy as a function of the bunch number for the first 10000 shots of our PCA test data set of 90000 pulse trains of 80 bunches each. The data are taken from a measurement at 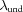 = 52 nm with argon as a target gas in OPIS. The decrease of the photon energy within the first ten bunches is caused by ion space charge of previous pulses.

In order to test the PCA method for OPIS operation we chose a measurement at 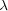 = 52 nm with argon as the target gas, hence having simple eTOF spectra which show just the Ar 3*p* photoemission line. The data set comprised 90000 FLASH2 pulses of 80 bunches. Fig. 9 ▸ shows the photon energy for the partition of the first 10000 shots of these data, derived in a post-recording offline analysis using the standard wavelength determination procedure based on a line profile fitting procedure. Moreover, a moving average scheme has been applied, in which mean spectra from a moving average of 21 single-shot spectra 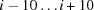 were used to determine the photon energy attributed to any single shot *i*. The results suggest a decrease in the photon energy of 0.28 eV within the first ten bunches of the pulse train which reflects a clear space charge effect.

Applying an adapted PCA software code, the basis elements have been derived from the partition of the first 10000 pulses. Each basis function has the form of one-bunch spectra of 3500 samples and one basis set has been determined for each bunch position *b* and each of the four eTOF spectrometers *e*. As a result we found that, apart from the respective mean trace 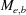 of the 10000 FEL shots, 15 principal basis traces 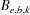 are sufficient for a reliable reconstruction 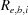 of a single-shot spectrum of pulse *i*, after calculation of the respective coefficients 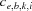. In terms of data storage, apart from saving the basis elements once which in this example comprises 4 × 80 × 16 × 3500 samples, in the running procedure only 15 coefficients per single-shot spectrum had to be stored instead of 3500 sampling points, which corresponds to a data reduction factor of more than 200.

**Figure 10:**
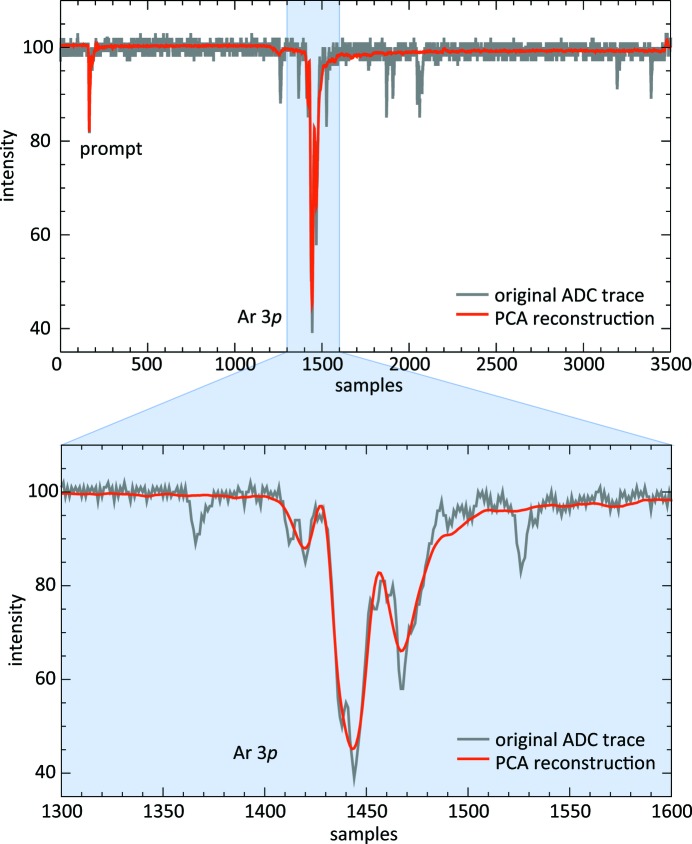
Comparison of a raw ADC trace of a single-shot spectrum of a certain bunch number in the pulse train with its reconstruction derived from the PCA analysis. The data are from a measurement with argon at 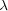 = 52.0 nm with pulse trains of 80 bunches. The upper panel shows the full TOF range of the spectrum block of bunch number 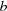 = 21, whereas a TOF interval around the Ar 3*p* photoemission line is magnified in the lower panel. The spectrum belongs to the first block of 10000 shots which were used for PCA basis determination.

In a first stage of the test, the spectra reconstruction within the partition used for basis determination has been evaluated. The two panels of Fig. 10 ▸ depict a showcase comparison of an original single-shot raw trace and the reconstruction of a spectrum. The noise is significantly reduced by the procedure and random spikes are eliminated. On the other hand, the major structure within the Ar 3*p* photoemission line is preserved and thus maintains the possibility for FEL spectral distribution analysis.

The difficulty when truncating the expansion in the PCA basis is to decide how many of the first *N* basis vectors need to be included, or from which point noise and artifacts dominate further components. We find that 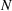 = 15 components results in a credible reconstruction in this case. Variances found for 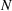 > 15 are substantially smaller than those in the leading components, so no significant loss of information is expected from this choice. However, we need to point out that this criterion is a rather soft one.

**Figure 11:**
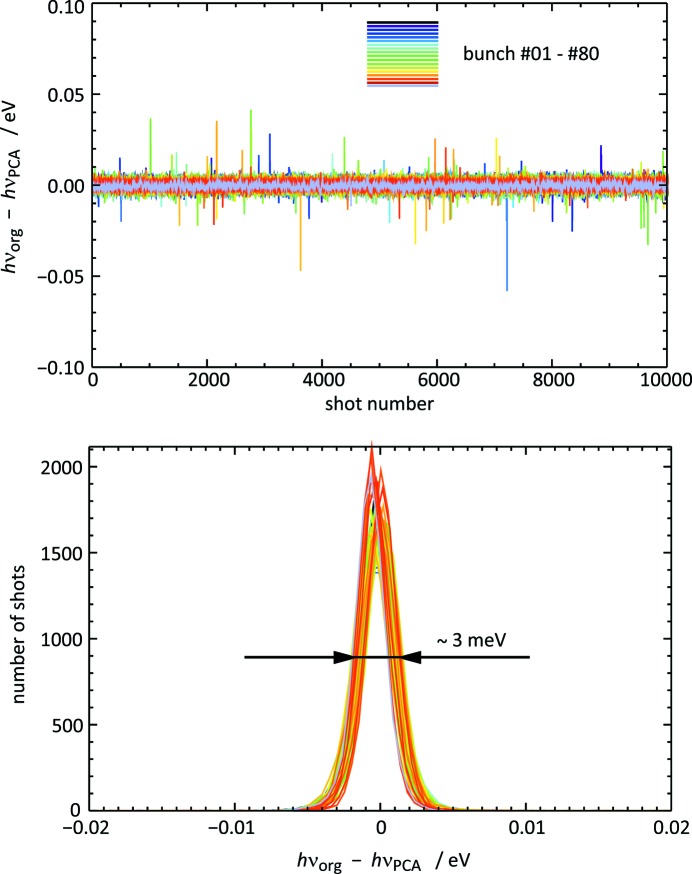
Differences 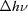 of the values of the photon energy 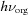 and 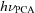 which have been derived by applying the standard wavelength monitoring analysis based on a line profile fit procedure to the original raw ADC traces and the reconstructed traces from the PCA analysis, respectively. The photon energy result for a single shot *i* was determined from a moving average spectrum of the 21 shots [*i* − 10, *i* + 10]. Upper panel: 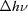 of 9992 single shots for all bunch numbers. Lower panel: histograms of 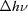 for every bunch number.

Secondly, we assessed the PCA-processed data in terms of their fidelity in wavelength monitoring. To this end, the same analysis software as used for the online wavelength monitoring including the moving average scheme described above was applied to the reconstructed traces. The difference between the two sets of results is shown in Fig. 11 ▸ by curves for each bunch position, in the form of a history plot and a histogram. The difference is well below the benchmark number of 0.1 eV concerning the absolute photon energy accuracy, *cf.* §2.2. In fact, the width of the histograms is only approximately 3 meV. Within the machine precision, the Pearson’s correlation coefficient between the two sets of photon energy results is equal to 1!

We will demonstrate now that the basis derived from the first 10000 shots can be generalized to represent also subsequent partitions with only an insignificant reduction of the quality of reconstruction. The reusability of a PCA basis is a necessary prerequisite to establish true online data processing where it is crucial to have a suitable basis at hand at the time when a spectrum is recorded.

We use the average RMS deviation of 10000 raw spectra and their reconstruction as a figure of merit *R* to decide whether the PCA basis set needs an update because the FEL beam parameters were tweaked or the settings of the diagnostics unit have changed, *i.e.* in *stationary* operation. For a direct comparison, we assess the 10000-shot partitions of the above-mentioned data set and derive 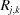, *j*, *k* = 0, 1,…, 8, *i.e.* *R* using the points from partition *j* projected onto the basis determined from the points in partition *k*. For 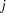 = 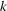, we call the basis intrinsic.

**Figure 12:**
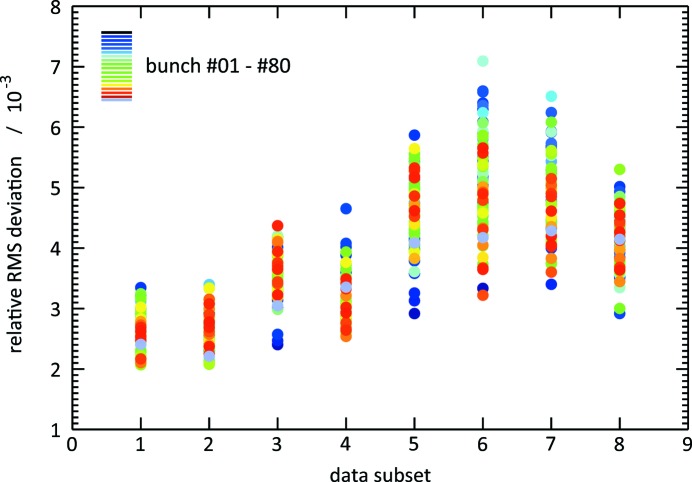
The RMS deviation between a raw ADC trace and its reconstruction, averaged over a partition of 10000 shots, is used to assess the overall quality of reconstruction. The relative RMS deviation 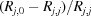 shown in the figure results from a direct comparison between the quality obtained when using the intrinsic basis of partition *j* with the one obtained from reusing the basis of partition 0.

Fig. 12 ▸ depicts the normalized differences 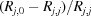 as a function of *j* for all of the 80 bunches in a train. It can be clearly seen that the raw data are reproduced except for an RMS residual which is typically only a few per mille larger than in the case of a reconstruction from its intrinsic basis. We attribute the trend observed in the shown result to long-term drifts and non-stationarities of the FEL which will potentially render a basis invalid after an extended period of time. We conclude from these results that *stationary* operation of the FEL and the spectrometer is in principle sufficient to allow for online PCA analysis of single-shot spectra.

Currently a wavelength analysis in real time for complete bunch trains is hindered due to the time-consuming fit algorithms used in the monitoring software. Least-squares fitting of an analytical model of the photoemission line has obvious advantages in the analysis of the instrument performance, concerning comparing peak positions in calibration procedures and investigations of spectral and detector response distributions. A way to overcome its computational downsides is to base the analysis on the momenta of the observed intensity distribution instead, as they can be computed explicitly without an iterative algorithm. This makes the evaluation not only much faster but also gives the procedure a deterministic run time. Here, the center of gravity as well as higher momenta of the photoemission peaks can be directly computed from the PCA coefficients without the need for an explicit reconstruction, again reducing the required computational power. Moreover, the future migration of the analysis to FPGA hardware becomes much more straightforward.

**Figure 13:**
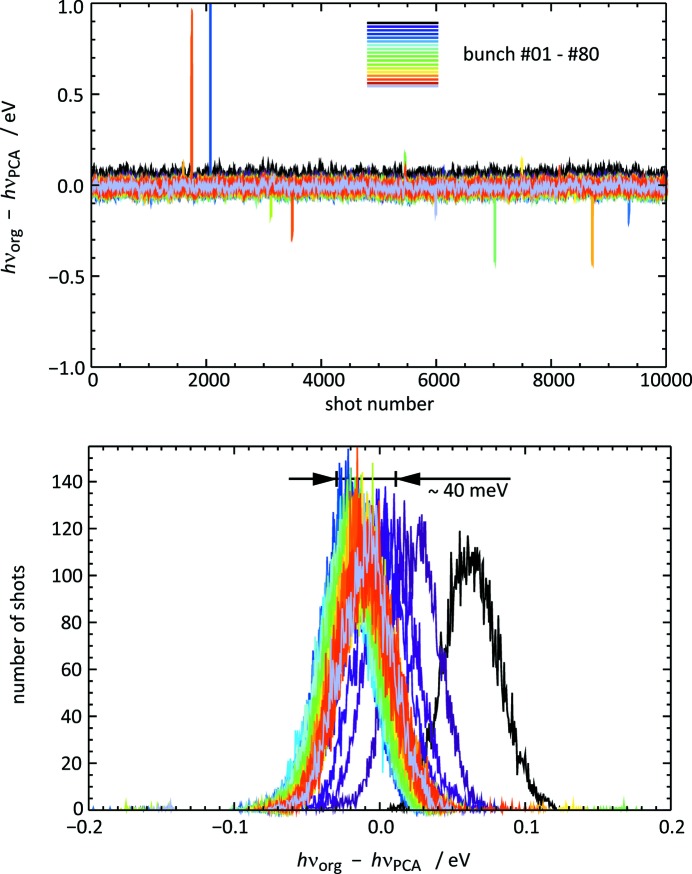
Differences 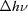 of the values of the photon energy 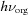 and 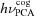. 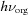 has been derived by applying the standard wavelength monitoring analysis based on a line profile fit procedure to the original raw ADC traces. 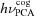 has been calculated converting the TOF values determined by center of gravity positions of the prompt signal and the Ar 3*p* photoemission lines with the existing time-to-energy calibration function. The photon energy result for a single shot *i* was determined from a moving average spectrum of the 21 shots [*i* − 10, *i* + 10]. Upper panel: 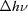 of 9992 single shots for all bunch numbers. Lower panel: histograms of 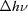 for each bunch number. Note that the plot ranges for 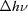 are ten times larger compared with Fig. 11 ▸.

So, as the final part of our studies testing the PCA method we calculated the centers of gravity of the prompt signal 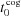 and the Ar 3*p* photoelectron line 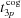 from the PCA coefficients for each single-shot spectrum of the test data set. The photon energy was derived by conversion of the corresponding TOF values 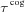 = 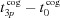 with the existing time-to-energy calibration function derived from peak positions. Again, this PCA method has been compared with the original wavelength analysis method for the first 10000-shot partition of the test data set, using the moving average scheme of 21 shots described above. Fig. 13 ▸ illustrates the result in the same way as Fig. 11 ▸. Here, the differences are much larger compared with the profile fit procedure, but are overall still within ±0.1 eV apart from a few outliers. The widths of the distribution of 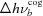 for individual bunches is of the order of 40 meV. In addition, the deviation of the mean wavelength of the bunches shows a trend along the bunch number which resembles the shifts caused by the space charge effect depicted in Fig. 9 ▸. The mean deviation ranges from about +0.06 eV for bunch 1 to about −0.02 eV for bunch numbers between 10 and 20. The calculated value of Pearson’s correlation coefficient is 0.88.

Comparing the results of wavelength determination using PCA shown in Figs. 11 ▸ and 13 ▸, it becomes obvious that the definition of the ‘center’ which references the position of the photoelectron signals plays a crucial role. The flight time values derived with centers of gravity of the photoemission peaks reveal an asymmetric line-broadening effect caused or enhanced by the space charge which apparently has no detrimental effect on the wavelength determination with the current calibration functions based on peak positions. On the other hand, for asymmetric real spectral distributions, *i.e.* without space charge, secondary effects or detector artifacts, using centers of gravity may be the more appropriate measure of the mean wavelength value. More insights regarding this matter can be gained by data evaluation using a new set of calibration functions based on centers of gravity in the future.

## 5. Conclusion and outlook   

Since the middle of 2016, the OPIS instrument has been the standard diagnostic tool for wavelength measurement at FLASH2 and has been routinely used in runs of user experiments as well as periods of internal studies. It features practically full photon transmission over the complete wavelength range. There are no optical elements involved which would have to be mechanically aligned or moved on wavelength changes.

The wavelength scale for OPIS measurements has been determined in a self-calibration procedure. As pointed out in this paper, the absolute uncertainty regarding the photon energy is of the order of 0.1 eV independently from the wavelength. This benchmark figure of accuracy has been achieved from calibration measurements which still include all stochastic fluctuations of the SASE-FEL FLASH2, even though optimized operation parameters and averaging were used in these measurements. On the other hand, in a user operation run, requirements for a successful experiment, such as for example the demand for high photon pulse energy and consequently large aperture sizes, sets boundary conditions which currently can be limiting for the OPIS performance. In that respect, the effect of space charge has been reported here.

In the basic operation mode the actual wavelength of a single selected bunch of the pulse train is monitored using real-time analysis in a moving average scheme of latest FEL shots. With the moderate computing power employed so far, the analysis-procedure-based iterative fit algorithms cannot fully keep pace with the FLASH repetition rate of 10 Hz. Even though the current online analysis speed is completely sufficient to cover enough data to deliver reliable results for monitoring purposes, it is mandatory to acquire data without gaps to deliver single-shot resolved information for user experiments. This is realised in the additional operation mode in which condensed photoelectron spectra data are stored online in the FLASH DAQ system. However, currently the analysis of full bunch trains has to be run offline.

One way to speed up the wavelength analysis is to boost the computing power, for example by employing GPU or FPGA hardware. This may be a suitable way to achieve full repetition rate in the monitoring mode. Another way is to replace the current fitting procedures by simpler algorithms, which is presumably inevitable aiming for real-time analysis of long bunch trains. The implementation of PCA in the data acquisition process offers the possibility to apply a combination of both methods. In a test example presented here we showed that with PCA a high data reduction can be achieved, maintaining at the same time sufficient details in the spectra reconstruction for evaluation of spectral distribution and shot-to-shot changes.

Apart from being used for wavelength monitoring as demonstrated in this paper, photoelectron spectroscopy is also applied in other fields of FEL diagnostics. As mentioned in the *Introduction* [Sec sec1], setups employing a number of eTOFs at various angles enables measurements of the polarization (Buck *et al.*, 2012 ▸; Allaria *et al.*, 2014 ▸; Lutman *et al.*, 2016 ▸). Moreover, temporal information such as the FEL pulse duration and arrival time can be determined with techniques involving streaking of photoelectrons by an external THz field (Grguraš *et al.*, 2012 ▸). The latter scheme is currently implemented at FLASH (Ivanov *et al.*, 2018 ▸). Here, the temporal information is projected onto the electron kinetic energy scale. For short pulse durations ≲20 fs, other approaches use intense circularly polarized laser sources to convert temporal information to angular space (Hartmann *et al.*, 2017 ▸).

The envisioned design upgrades of FEL sources towards continuous-wave (CW) operation requires a reassessment of gas-based diagnostic instruments. Recent test measurements with the GMD intensity monitor show that target depletion in CW schemes may cause errors of several 10%. For OPIS wavelength measurements, again the influence of ion space charge is the major challenge in that respect, depending on the intended CW repetition rate and the achievable pulse energy. On the technical side, we expect no loss of gain or current limit excess for MCP electron detectors in scenarios comparable with the FLASH2 repetition rates of 1 MHz and pulse energies of a few hundred microjoules. Certainly, such an upgrade would considerably increase the challenges regarding data acquisition and handling.
